# Evaluating Human Immune Responses for Vaccine Development in a Novel Human Spleen Cell-Engrafted NOD-SCID-IL2rγNull Mouse Model

**DOI:** 10.3389/fimmu.2018.00601

**Published:** 2018-03-23

**Authors:** Stéphanie Ghosn, Soulaima Chamat, Eric Prieur, Antoine Stephan, Pierre Druilhe, Hasnaa Bouharoun-Tayoun

**Affiliations:** ^1^Laboratory of Immunology and Vector Born Diseases, Faculty of Public Health-Fanar, Lebanese University, Beirut, Lebanon; ^2^Vac4All Initiative, Paris, France; ^3^Faculty of Medicine, Lebanese University, Hadath, Lebanon; ^4^National Organization for Organ and Tissues Donation and Transplantation (NOOTDT), Beirut, Lebanon

**Keywords:** humanized mouse model, vaccine development, *Plasmodium falciparum*, human spleen cells, malaria, immunogenicity, immunocompromised mice

## Abstract

The lack of preclinical models able to faithfully predict the immune responses which are later obtained in the clinic is a major hurdle for vaccines development as it increases markedly the delays and the costs required to perform clinical studies. We developed and evaluated the relevance to human immune responses of a novel humanized mouse model, humanized-spleen cells-NOD-SCID-gamma null (Hu-SPL-NSG), in which we grafted human spleen cells in immunodeficient NOD-SCID-IL-2rγnull (NSG) mice. We selected the malaria vaccine candidate, Liver Stage Antigen 3-Full Length, because we had previously observed a major discrepancy between preclinical and clinical results, and compared its immunogenicity with that of a shorter form of the molecule, LSA3-729. NSG mice engrafted with human spleen lymphocytes were immunized with either LSA3-FL or LSA3-729, both adjuvanted with montanide ISA720. We found that the shorter LSA3-729 triggered the production of human antibodies and a T-helper-type 1 cellular immune response associated with protection whereas LSA3-FL did not. Results were consistent in five groups receiving lymphocytes from five distinct human donors. We identified antigenic regions in the full-length molecule, but not in the shorter version, which induced T-regulatory type of cellular responses. These regions had failed to be predicted by previous preclinical experiments in a wide range of animal models, including primates. Results were reproducible using spleen cells from all five human donors. The findings in the Hu-SPL-NSG model were similar to the results obtained using LSA3-FL in the clinic and hence could have been used to predict them. The model does not present graft versus host reaction, low survival of engrafted B lymphocytes and difficulty to raise primary immune responses, all limitations previously reported in humanized immune-compromised mice. Results also point to the shorter construct, LSA3-729 as a more efficient vaccine candidate. In summary, our findings indicate that the Hu-SPL-NSG model could be a relevant and cost-saving choice for early selection of vaccine candidates before clinical development, and deserves being further evaluated.

## Introduction

The development of new medical products is a very long and costly process, fraught with uncertainty and risks. For vaccines, preclinical testing in animal models plays an essential role in this process, providing preliminary data on the immunogenic characteristics of the emerging vaccine concept as well as the preliminary proof of the targeted benefit. However, results are highly dependent on the relevance of available models. Selection of concepts, vaccine mechanisms, choice of antigenic regions, formulations as well as doses, schedules, and routes of administration are made mostly or only based on the immune responses these candidates induce in animal models. However, it has been widely acknowledged that the limitations of conventional animal models induce high uncertainties about the real outcome in the clinic; “mice lie” is an often cited phrase to caution against the over extrapolation of their results onto humans (1, 2). Despite this, they remain widely used for preclinical vaccine evaluation, likely because they are readily available, convenient and above all there are no alternatives with demonstrated superiority. The consequences of the dependence on such preclinical models can be quite substantial as poor choices could be made that would be conducted into long and costly clinical development that bears a high risk of failure.

This highlights the need for animal models predictive of human immune responses. The last 20 years have witnessed the development of immunodeficient mice that can support the survival of functional human cells and tissues in an attempt to create immunogenicity model systems, so-called “humanized mice” that reflect human responses more faithfully than conventional rodent models. These studies have benefited from numerous advances in the design of mice combining several genetic substitutions leading to stable immune deficiencies. Despite progress at the host animal level and in the type of human cells grafted, they still remain not fitted to the needs. We attempted to develop an improved model, based upon the engraftment of immunodeficient NOD-SCID-IL-2rγnull (NSG) mice with human spleen lymphocytes [humanized-spleen cells-NOD-SCID-gamma null (Hu-SPL-NSG)], and use it to evaluate the immune responses to malaria vaccine candidates. Our aim was to overcome limitations of other humanized mouse models, namely those that rely on engraftment of human peripheral blood mononuclear cells, hematopoietic stem cells, or bone marrow, fetal liver, and thymic cells. These limitations include poor human humoral immune responses, especially IgG, graft versus host disease, and technically demanding engraftment requirements (reliance on human fetal tissue or surgical grafting methods) [reviewed in Ref. (3–8)]. In contrast, human spleen cells which contain many discrete cell subsets, particularly dendritic cells, had been found of value to induce the production of human antibodies in SCID mice (9–11) or to study the response to human tissue grafted in Scid-beige mice (12–16). This led us to perform a systematic comparison of PBMC versus spleen cells which showed that spleen cells were superior (Ghosn S. et al., manuscript in preparation). In the present study, we combined progress in mice deficiencies with the use of human spleen cells, as a model to assess the human immunogenicity of novel vaccines.

**Figure 1:**
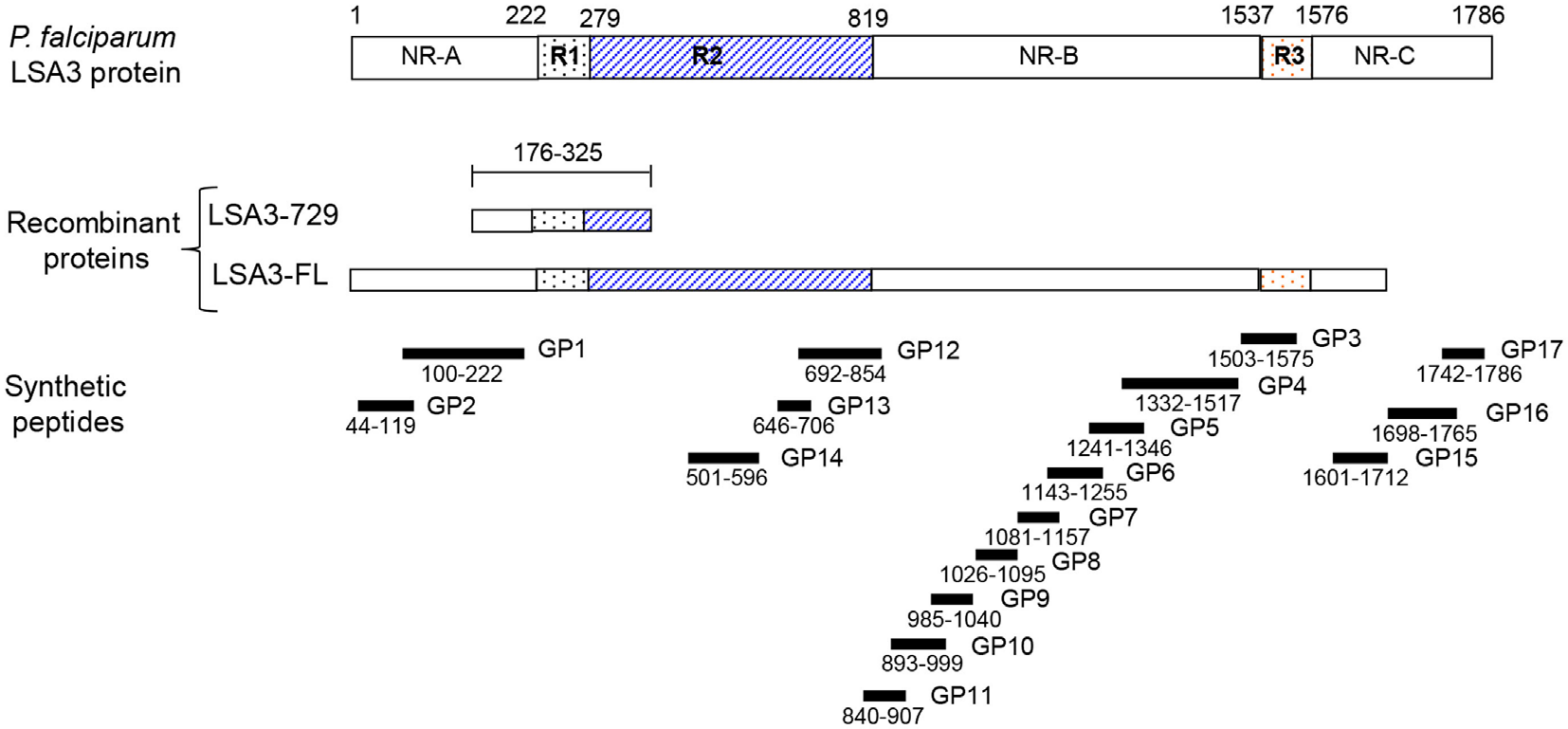
Schematic representation of liver stage antigen 3-full length (LSA3-FL) and liver stage antigen 3-729 (short form) (LSA3-729) constructs derived from *Plasmodium falciparum* liver stage antigen 3. Shown is the localization of the repetitive (R1, R2, R3) and non repetitive (NR-A, NR-B, NR-C) regions, the synthetic peptides (LSA3-GP). The numbers indicate amino acid positions in 3D7 strain.

We selected two malaria vaccine candidates because they were best fitted to our study objective. Both are based on the *Plasmodium falciparum* liver stage antigen 3 (LSA3) that induced T-regulatory cells only in humans, which failed to be detected in animals. This antigen has been previously described as a promising vaccine candidate against malaria (17). This protein is expressed both on the surface of the parasitic sporozoite stage and on infected human hepatocytes. It was identified through recognition of a short fragment of the protein (the clone LSA 729) by sera of subjects who were protected against malaria challenge following immunization with irradiated sporozoites (17), suggesting that liver stage antigen 3-729 (short form) (LSA3-729) (see Figure 1) is a target of protective immune responses. The antigenic properties of LSA3 have been shown in sera from malaria-exposed populations using short and long peptides spanning the entire length of the large LSA3 molecule (17–20). Various constructs derived from different regions of the molecule found to be most antigenic have been tested in a variety of formulations, in chimpanzees (17, 21), aotus monkeys (22), and mice (19, 23, 24), and found to be immunogenic. Proof-of-concept of the protective potential of LSA3 has been demonstrated in the same models, most convincingly in chimpanzees, where vaccination induced protection against massive and multiple *P. falciparum* sporozoite challenges. However, protection studies were performed using formulations mostly derived from the originally recognized short LSA3 protein fragment, LSA3 729 (Figure 1) (17, 25), whereas in the first in-human trial, the much larger full length LSA3 (LSA3-FL) protein was tested, expressed as a recombinant protein, LSA3-FL. Formulated in either aluminum hydroxide or montanide ISA720, LSA3-FL, was immunogenic in mice, rats, macaques, aotus monkeys (see Figure S1 in Supplementary Material) and, noticeably, induced in these animal models strong antigen-specific IFN-γ responses, which have been identified as a potential surrogate of protection in *P. falciparum* sporozoite challenge studies (26). In contrast, while it was safe in human adult volunteers, LSA3-FL elicited a very unusual profile of responses: in a three-dose vaccination schedule given 28 days apart, the first and second vaccination induced only a modest rise of antigen-specific IFN-γ and antibody responses, whereas the third vaccination induced a marked decrease in IFN-γ responses to preimmunization levels, and a modest and transient rise in antibodies followed by a drop to pre-3d immunization titers at day 140 (see Figure S2 in Supplementary Material). Detailed analysis of immune responses in volunteers using 17 long peptides spanning the whole 220 kDa protein (Figure 1) highlighted the presence of T regulatory (Treg) sequences outside the LSA3-729 region which triggered the secretion of IL-10 (see Figure S3 in Supplementary Material).

We capitalized on the major discrepancy between results obtained in the clinic and in animals to evaluate the relevance of our immunogenicity model. We analyzed in detail the immune response elicited by LSA3-FL in Hu-SPL-NSG mice and compared it to that induced in the clinical trial participants. In order to improve vaccine construct, we further compared the immunogenicity of LSA3-FL with that of the shorter form of the molecule, LSA3-729 (Figure 1). Human splenocytes derived from five different donors, with distinct HLA backgrounds, were engrafted in 10 groups of NSG mice and were immunized with either LSA3-FL or LSA3-729, both formulated with montanide ISA720. Human B and T cell immune responses were assessed by measuring (i) total human IgG by ELISA, (ii) LSA3-specific antibody responses by ELISA, (iii) T-cell responses by enzyme-linked immunospot (ELISPOT), and (iv) a panel of cytokines and chemokines determined by quantitative reverse transcription PCR, chosen to differentiate T-cell subsets, particularly T-helper and T-regulatory cells.

Responses in the Hu-SPL-NSG mice corroborated the results in humans immunized with LSA3-FL, with induction of Treg secretion patterns, indicating that the model was able to faithfully reflect the immune responses we recorded in trial participants, not predicted by previous results in the conventional animal models. In addition, the results confirmed the immunogenicity of LSA3-729, previously shown to induce protection against *P. falciparum*, and therefore the value of LSA3-729 for future clinical studies. Our data document the Hu-SPL-NSG model as a valuable preclinical model for vaccine development.

## Materials and Methods

### Ethical Considerations and Human Spleen Donation

Approval for this study was obtained from the ethical committee of the National Organization for Organ & Tissue Donation & Transplantation (NOOTDT) in Lebanon. NOOTDT is a nonprofit national organization affiliated to the Lebanese Ministry of Health. It has the exclusive responsibility of supervising organ and tissue donation for medical purposes and scientific research. The human spleens were obtained from deceased organ donors. Informed consent to donate organs for transplantation and scientific research was obtained by NOOTDT from the donors or their relatives.

### Human Spleen Cell Preparation

Human spleens were processed within 24 h after surgical excision as previously described (9). Briefly, splenic tissue was dissected and a cell suspension was prepared. Red blood cells were lyzed using Gey’s solution for 5–10 min at 25°C. After washing, leukocytes were resuspended in medium consisting of 37% fetal calf serum (Sigma, USA), 10% dimethyl sulfoxide (Sigma, USA), and 53% RPMI1640 (Sigma, USA), and then cryopreserved in liquid nitrogen until used. The mean number of cells isolated from each spleen donor was 10 ± 4 billions.

### Animals

NOD.Cg-Prkdc^scid^-IL2rg^tm1WjI^/SzJ (NSG) mice were obtained from The Jackson Laboratories (USA) and housed in sterile microisolators. All food, water, caging, and bedding were autoclaved before use. The 6- to 8-week-old NSG mice were included in the experiments. Due to the absence of legislation and guidelines for the use of experimental animals in Lebanon, animal handling and experiments were conducted by trained personnel in accordance with the “Guide for the care and use of laboratory animals; eighth edition,” The National Academies Press, Washington DC, USA.

### Antigens

The amino acid sequences of the recombinant proteins and peptides employed in this study correspond to the *P. falciparum* LSA3 of the 3D7 reference parasite strain (1558 AA) (PlasmoDB accession number; PF3D7_0220000). The recombinant protein LSA3-FL (aa 89–1,414) has been produced in the bacteria *Lactococcus lactis* and purified to clinical grade batches by HENOGEN (Brussels, Belgium). Immunization of three groups of rats with recombinant protein LSA3-FL, adjuvanted with montanide ISA720, or alum, or without adjuvant, induced a rapid and strong specific antibody response after 3 immunizations in all animals in pharmacotoxicological studies performed by Confarma (Figure S1 in Supplementary Material). The recombinant protein LSA3-729 (aa 176–325) was produced as a histidine tailed fusion protein as previously described (17). Synthetic peptides LSA3-GP2 (aa 44–119), LSA3-GP4 (aa 1,332–1,517), and LSA3-GP8 (aa 1,026–1,095) were produced by Dr. G.P. Corradin as previously described (19, 20).

### Engraftment and Immunization of Human Spleen Cells in NSG Mice

Human spleen cells were primed *in vitro* prior to engraftment according to a previously reported protocol (9) with modifications. Cells were cultured on day 0 at 4 × 10^6^ cells/ml with or without 1 µg/ml of antigen in RPMI1640 medium supplemented with 10% FCS, 1% non-essential amino acids (100×), 2 mM glutamine, 2 mM sodium pyruvate, and 50 µg/ml gentamicin (complete medium). All culture reagents were purchased from Sigma, USA. On day 1, recombinant human IL-2 (Gibco Invitrogen) was added at 25 IU/ml. On day 3, the spleen cells were harvested, washed and resuspended in Hank’s Balanced Salt Solution (HBSS). Each NSG mouse received an intraperitoneal injection (ip) of 30 × 10^6^ antigen-primed or unprimed spleen cells. At day 7 and day 21, reconstituted mice (Hu-SPL-NSG) were boosted with 10 µg of antigen injected intraperitoneally (ip) in 200 µl of HBSS-montanide ISA720 adjuvant (v/v). Mice reconstituted with unprimed spleen cells, received only adjuvant. Blood samples were collected 1 week after each booster.

### Determination of Human Total IgG and Antigen-Specific Antibodies in Hu-SPL-NSG Sera

These determinations were performed by ELISA. Total human IgG was measured in each mouse serum in order to indirectly assess human cell engraftment and monitor that human lymphocytes kept physiological functions. Briefly, for the detection of total human IgG in mouse serum, flat-bottomed microtitration plates (Nunc-Thermo Scientific, USA) were coated overnight at 4°C with 2 µg/ml purified goat anti-human IgG (H + L) (Invitrogen, USA) in 0.1 M carbonate buffer, pH 9.5. After washing (PBS, pH 7.2) and saturation (PBS, 3% non-fat milk), test sera (diluted in PBS, 3% non-fat milk, 0.05% Tween20) were added and incubated for 1 h. Negative controls consisted of preimmune mouse serum of the same animals. Human IgG were revealed by subsequent addition of horseradish peroxidase-conjugated goat anti-human IgG (H + L) (Invitrogen, USA) for 1 h, followed by the peroxidase substrate Tetramethylbenzidin (Amresco, USA). Total human IgG concentration in the Hu-SPL-NSG mouse serum was calculated in comparison with a standard human IgG solution (Zymed, USA). The same test was performed for the detection of antigen-specific antibodies, except that plates were coated with the corresponding antigen at 2.5 µg/ml in PBS, pH 7.2. In this case, negative controls consisted of sera of individuals that have never been infected with *Plasmodium*, while positive controls consisted of a pool of sera from hyperimmune African adults. In both cases, serial dilutions of each serum were tested in order to determine antibody titer. For specific antibody titer determination, a test was considered positive when it yielded an absorbance above cutoff (negative control OD × 2).

### Assessment of Human Cell Homing in Hu-SPL-NSG Mice

The Hu-SPL-NSG mice were sacrificed on day 28 and for one preliminary experiment, cell suspensions were prepared from the peritoneal cavity, peripheral blood and spleen. As human cells were found to home predominantly to the spleen, for the remaining experiments only spleen cells were analyzed. After washing, cell suspensions were incubated for 1 h on ice with mouse antibodies specific for human surface antigens CD45 (leukocyte marker) (Abcam, UK) or CD3 (T cell marker) (Abcam, UK) diluted in HBSS containing 1% bovine serum albumin (Sigma, USA). Goat antimouse coupled to fluorescein isothiocyanate (Invitrogen) was used as secondary antibody. The percentage of fluorescent human leukocytes was evaluated by fluorescence microscopy.

### Evaluation of Human Cellular Response by ELISPOT Assay

Enzyme-Linked Immunospot 96-well plates were coated overnight at 4°C with 5 µg/ml monoclonal antibodies specific for human cytokine IFN-γ, IL-2, or IL-10. The spleen cells of each group of immunized and non immunized Hu-SPL-NSG mice were pooled and added at 3 × 10^5^ cells per well. Cells in triplicate wells were incubated for 36 h at 37°C, 5% CO_2_, with the immunizing antigen (5 µg/ml), concanavalin A (Con A, 5 µg/ml) (Sigma, USA), or culture medium alone. Wells were then washed twice with deionized H_2_O and three times with PBS, 0.05% Tween20 before adding the corresponding biotinylated anti-human cytokine antibody for 2 h at room temperature. After washing, streptavidin conjugated to horseradish peroxidase diluted 1/100 was added for 1 h. The spots were visualized using the horseradish peroxidase substrate 3-amino-9-ethylcarbazole. Plates and reagents were purchased from BD Biosciences, USA.

### Quantification of Gene Expression of Different Cytokines, Chemokines and Transcription Factors by Real-Time Reverse Transcription PCR (RT-qPCR)

Spleen cells of each group of immunized and non immunized mice were pooled and cultured at 2 × 10^6^ cells/ml in complete RPMI1640 medium, with or without stimulation with the immunizing antigen. Total cellular RNA was extracted after a 24 h culture using RNeasy Mini Kit (Quiagen, Germany). Reverse transcription of 400 ng of total RNA was carried out using RevertAid M-MuLV enzyme (RevertAid kit First Strand Synthesis kit, Thermo Scientific Fermentas) in a 20 µl reaction mixture. Quantitative PCR was used to measure the relative expression of different human cytokines, chemokines and transcription factors considered as characteristic for Th1 or Treg cells. The PCR mixture was performed using LightCycler 480 SYBR Green Master, La Roche as recommended by the manufacturer. The TGFβ1, CCL22, FOXP-3 and T-bet primers were designed using the program provided by the NCBI website (http://www.ncbi.nlm.nih.gov/tools/primer-blast/) while the IL-10 and CXCL10 primers were already published (27, 28). Primers sequences are presented in Table 1. Primers specificity was confirmed by agarose gel electrophoresis. PCR amplifications were performed at 95°C for 5 min (1 cycle), followed by 45 cycles of incubation at 95°C for 10 s and 55–57°C for 10 s in the LightCycler 480 machine (La Roche). Each sample was run in triplicate. The housekeeping genes used as internal references were beta-actin (β-actin) and hypoxanthine phosphoribosyltransferase 1 (geNorm analysis). The cycle threshold (Ct) value was calculated based on the produced PCR curve. The relative expression levels were calculated by the 2^ΔΔCt^ method.

**Table 1:** List of primers specific for human genes used in qPCR experiments.

Human gene	Forward primer	Reverse primer
FOXP-3	5′-ACCTGTGGGGTAGCCATGGAA-3′	5′-TTGTGGCGGATGGCGTTCTT-3′
IL-10	5′-ACCTGCCTAACATGCTTCGAG-3′	5′-CTGGGTCTTGGTTCTCAGCTT-3′
TGF-β1	5′-GTCTGCTGAGGCTCAAGTTA-3′	5′-GATGTCCACTTGCAGTGTGT-3′
CCL22	5′-GACTGCACTCCTGGTTGTCCT-3′	5′-AGCAGACGCTGTCTTCCATGT-3′
T-bet	5′-CTATCCTTCCAGTGGTGACA-3′	5′-CACTTCCCCAACCAACTACT-3′
CXCL10	5′-CTGTACGCTGTACCTGCATCA-3′	5′-GGAGATCTTTTAGACCTTTCCTTG-3′

### Statistical Analysis

The nonparametric Mann–Whitney test was used to compare the results between different experimental mice groups. Statistical significance was determined at *p* < 0.05.

## Results

### LSA3-729, but Not LSA3-FL, Consistently Induces Human-Specific Antibodies in Hu-SPL-NSG Mice

Liver stage antigen 3 is expressed in liver forms and on the surface of sporozoites. Anti-LSA3 antibodies can prevent invasion of hepatocytes by sporozoites (29), and hence are contributing to protection. The two different LSA3 constructions, the LSA3-FL and the shorter one LSA3-729 (aa 176–325) (Figure 1), were first compared for their ability to induce a human antibody response.

**Figure 2:**
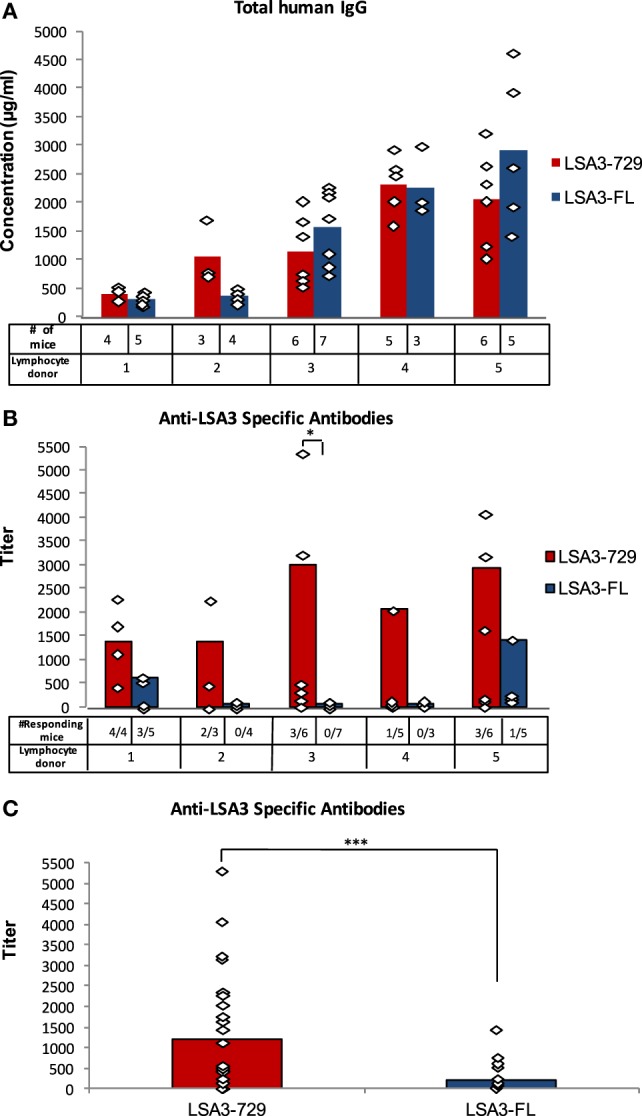
Human B cell responses elicited by liver stage antigen 3-full length (LSA3-FL) and liver stage antigen 3-729 (short form) (LSA3-729). **(A)** Total IgG and **(B,C)** LSA3-specific antibodies. NSG mice were engrafted with *in vitro* primed human spleen cells obtained from five different donors (1–5). Humanized-spleen cells-NOD-SCID-gamma null (Hu-SPL-NSG) mice were immunized with either LSA3-FL (LSA3 full length) or LSA3-729, a markedly shorter region of the LSA3 antigen, at days 7 and 21. The number of mice in each group is presented in panel **(A)**. Sera were collected on day 28 and tested by ELISA for the detection of total human IgG **(A)** and of LSA3-specific antibodies **(B,C)**. The concentration of total human IgG in the Hu-SPL-NSG mice sera was calculated using the results of standard human IgG included as positive control in each experiment. The adjusted specific antibody titer was calculated using the following formula: (antigen-specific antibody titer/total human IgG concentration) × 10 in order to normalize individual antibody responses by 10 mg, and thereby allow to compare animals in this respect. A specific antibody titer is considered positive when it is higher than the mean + 2 SD of non immunized mice sera titer. The number of antibody responding mice in each group is shown in panel **(B)**. Data are presented as mean values observed in all immunized mice **(A,C)** or in responding mice **(B)**. Each dot corresponds to the result of one individual mouse in each experimental group. Statistically significant difference is indicated when applicable: *p* = 0.035 (*) and *p* = 0.016 (***).

Human spleen cells derived from five different donors were engrafted in five corresponding groups of NSG mice. Each of the five groups was divided into two subgroups, immunized with either LSA3-729 or LSA3-FL on days 7 and 21. One week after the third immunization, the concentration of total human IgG and the titer of human antibodies specific for the immunizing antigen were measured in the serum of each Hu-SPL-NSG mouse. Successful survival of functional human lymphocytes in each of the 10 subgroups of engrafted mice was indirectly demonstrated from the levels of total human IgG in mice sera. Peak levels varied from one spleen donor to the other, ranging from 400 to 2,900 µg/ml (Figure 2A).

Vaccination with the short LSA3-729 formulation induced specific antibody responses in mice engrafted with spleen cells from all five donors, whereas the large LSA3-FL was immunogenic in mice reconstituted with cells from only two out of five donors. The percentage of responding mice also differed; in the case of LSA3-729, 54% of immunized mice (13/24) produced specific antibodies, when compared with 16.7% of mice immunized with LSA3-FL (4/24) (Figure 2B). Comparing levels of induced specific antibodies, anti-LSA3-729 antibody titers were significantly higher than that of anti-LSA3-FL antibodies (*p* = 0.016) (Figure 2C).

These three parameters characterizing the LSA3-specific antibody response indicate that LSA3-729 is more immunogenic to human lymphocytes than LSA3-FL, suggesting that the large regions flanking the 176–325 aa sequence in the full-length protein may affect the quality and amplitude of human response to LSA3.

### Interferon-γ, the Surrogate Marker of Protection Against Malaria Liver Stages, Is Induced Only by LSA3-729

Protection against *P. falciparum* sporozoite challenges was found to be significantly associated with antigen-specific secretion of IFN-γ (26). In order to study the cellular immune responses in the Hu-SPL-NSG mice, we first assessed the homing of the engrafted cells. We found that, regardless of the donor or the nature of the antigen, human CD45^+^ cells migrate preferentially to the spleen where they represent approximately 40% of total cells on day 28 after engraftment (Figure S4 in Supplementary Material). Hence, we used the cells recovered from the spleens of Hu-SPL-NSG mice to study the human T cell response to the antigens *in vitro*.

**Figure 3:**
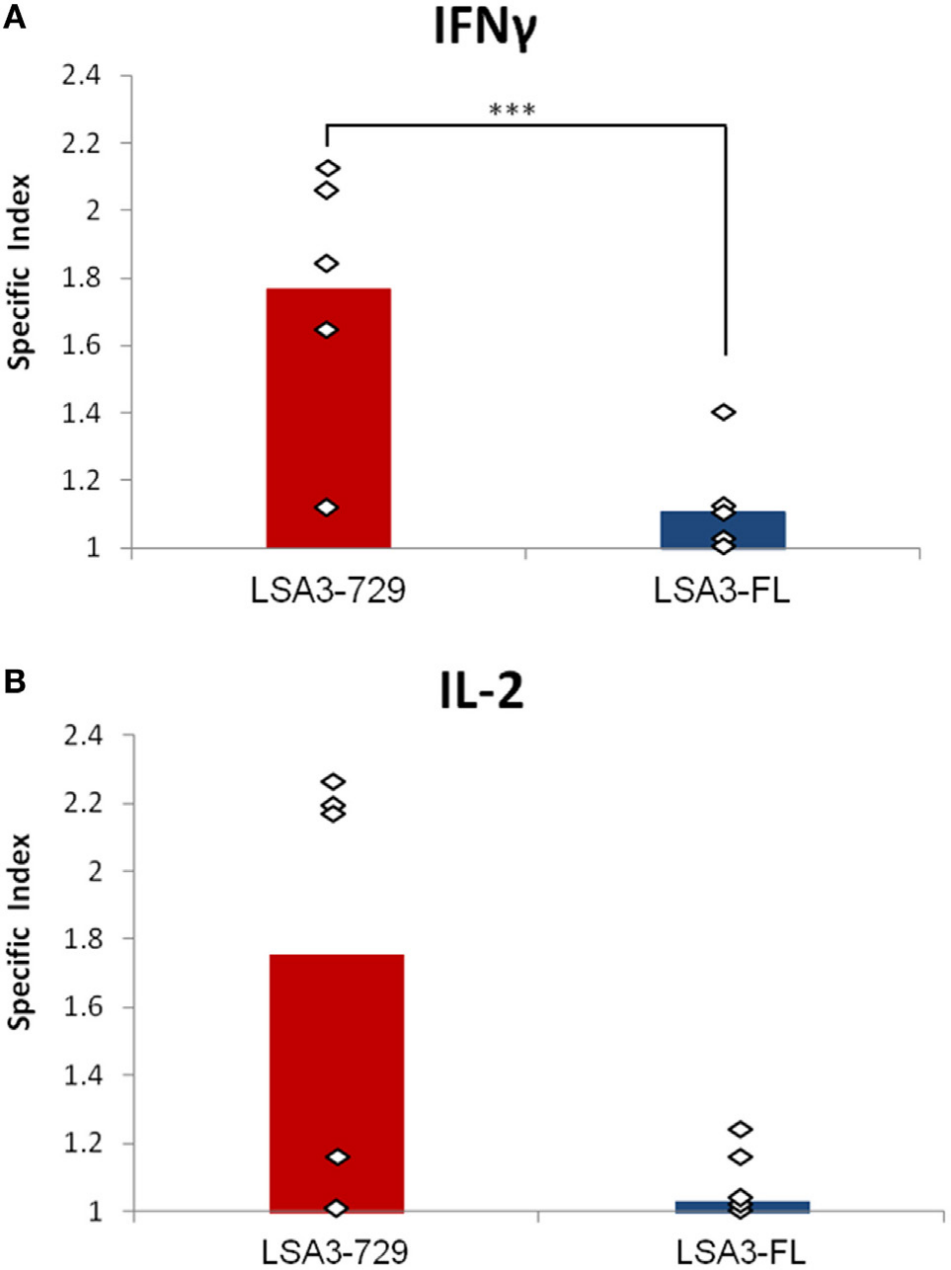
T cell-dependent IFN-γ and IL-2 responses elicited by liver stage antigen 3-full length (LSA3-FL) and liver stage antigen 3-729 (short form) (LSA3-729). NSG mice were engrafted with *in vitro* primed human spleen cells obtained from five different donors. The humanized-spleen cells-NOD-SCID-gamma null mice were immunized with LSA3-FL or LSA3-729 antigen at days 7 and 21. At day 28, spleen cells of each group of mice were recovered, pooled and cultured *in vitro* with or without antigen stimulation. Human IFN-γ **(A)** or IL-2 **(B)** production was measured by enzyme-linked immunospot. The specific index is the ratio of spot forming cells (SFC) of antigen stimulated cells over SFC of non stimulated cells. Data are presented as mean values of the results obtained with each immunizing antigen using human spleen cells from five different donors. Each dot corresponds to the result obtained with spleen cells pooled from mice of one experimental group. Statistically significant difference is indicated when applicable: *p* = 0.016 (***).

We investigated whether LSA3-729 and LSA3-FL are able to induce IFN-γ and IL-2 production in Hu-SPL-NSG mice. Spleen cells from immunized mice were cultured *in vitro* with or without the immunizing antigen, and human IFN-γ and IL-2 production was measured by ELISPOT. A significant difference in IFN-γ response was noted between the two vaccine constructs (*p* = 0.016). As shown in Figure 3, LSA3-729 induced specific production of both IFN-γ and IL-2 in spleen cells from mice reconstituted with splenocytes from 5/5 and 4/5 tested donors, respectively, whereas low to negative IFN-γ and IL-2 responses were detected in cells from mice immunized with LSA3-FL. These results, that are in-keeping with the specific antibody response (Figure 2), indicate that the shorter construction LSA3-729 can generate an immune response associated with protection against the preerythrocytic stages of *P. falciparum* whereas the entire protein does not.

### A T-Regulatory Response May Be Induced by LSA3-FL and Not by LSA3-729

Since the entire protein LSA3-FL was unable to induce immune responses associated with protection, in contrast to the shorter LSA3-729, we explored in greater detail the cellular immune response induced by each construction, and focused our assessment on Treg and T-helper type 1 cell (Th1) markers. For this purpose, we cultured spleen cells of Hu-SPL-NSG mice immunized with either LSA3-FL or LSA3-729 and measured by RT-qPCR the expression of Treg-related molecules, mainly IL-10 and TGF-β1 cytokines, CCL22 chemokine and FOXP-3 transcription factor; or the expression of Th1-related markers, namely the CXCL10 chemokine and the T-bet transcription factor.

**Figure 4:**
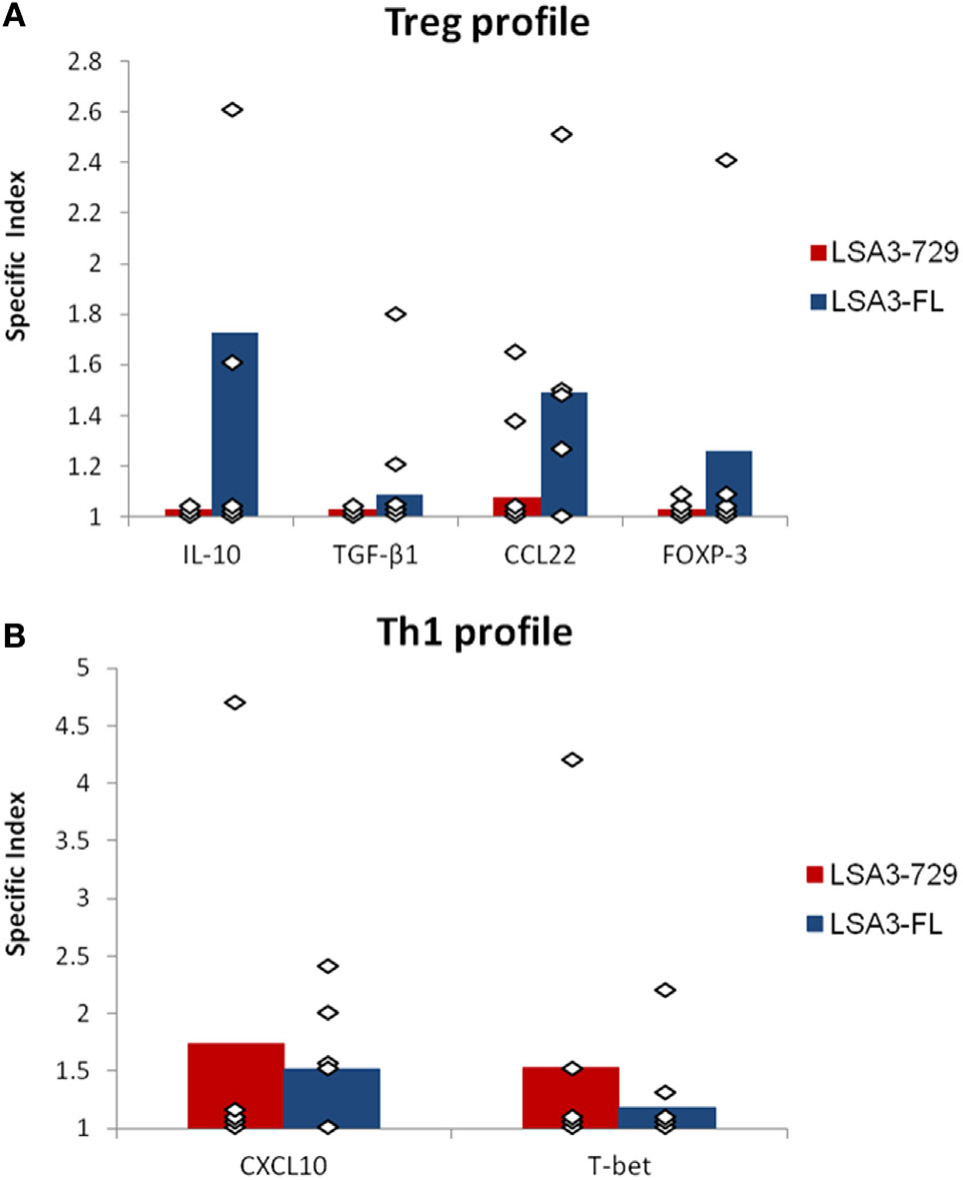
Evaluation of human T-helper type 1 (Th1) and T regulatory (Treg) responses elicited by liver stage antigen 3-full length (LSA3-FL) or liver stage antigen 3-729 (short form) (LSA3-729). At day 28 after immunization, humanized-spleen cells-NOD-SCID-gamma null (Hu-SPL-NSG) spleen cells were cultured *in vitro* with or without antigen stimulation. The expression of molecules characteristic of a Treg response (cytokines IL-10 and TGF-β1, chemokine CCL22, and transcription factor FOXP-3) **(A)** or a Th1 response (chemokine CXCL10 and transcription factor T-bet) **(B)** was measured by real-time reverse transcription PCR. The specific index is the ratio of normalized relative quantity (NRQ) of *in vitro* antigen stimulated cells over NRQ of non stimulated cells. Data are presented as mean values of the results obtained with each immunizing antigen using human spleen cells from five different donors. Each dot corresponds to the result obtained with spleen cells pooled from mice of one experimental group.

The results showed that LSA3-FL induced in several experiments simultaneous expression of IL-10, CCL22, and FOXP-3, while, except for a low production of CCL22, none of these markers of Treg activation was expressed by human cells harvested from Hu-SPL-NSG mice immunized with the small protein LSA3-729 (Figure 4A). As for the expression of Th1-related molecules, LSA3-FL induced the expression of low to moderate levels of CXCL10 and T-bet. In LSA3-729 immunized animals, the production of the transcripts from these two markers varied from one human cell donor to the other, possibly reflecting genetic differences in the ability to react with the corresponding epitopes (Figure 4B). Taken together, these findings indicate the presence of Treg inducing sequences in LSA3-FL though not in LSA3-729.

### The Model Highlights the Same Treg Sequences as Those Observed in Human Volunteers

**Figure 5:**
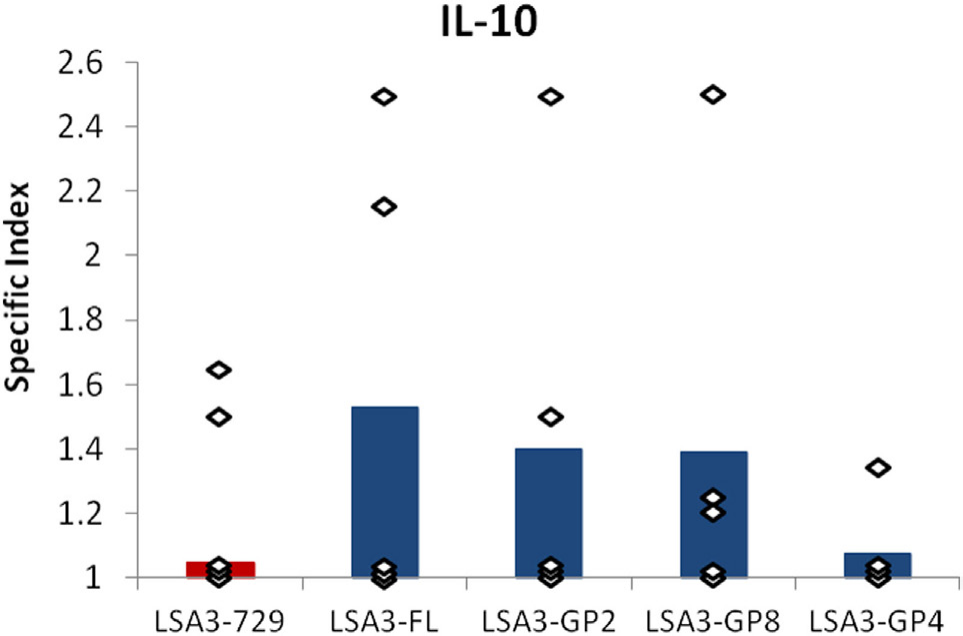
IL-10 production induced in spleen cells from liver stage antigen 3-full length (LSA3-FL) immunized mice by peptides LSA3-GP2 and LSA3-GP8. Spleen cells of the LSA3-FL immunized humanized-spleen cells-NOD-SCID-gamma null mice were stimulated *in vitro* with three synthetic peptides of LSA3 (LSA3-GP2, -GP8, and -GP4). The specific production of IL-10 was measured by enzyme-linked immunospot. The specific index is the ratio of spot forming cells (SFC) of *in vitro* stimulated cells over SFC of non stimulated cells. Data are presented as mean values of the results obtained with each stimulating antigen using human spleen cells from five different donors. Each dot corresponds to the result obtained with spleen cells pooled from mice of one experimental group.

Spleen cells from Hu-SPL-NSG mice immunized with LSA3-FL were stimulated *in vitro* with the LSA3-GP2 (N-terminal) or LSA3-GP8 (central region) peptide. These two peptides were previously identified among a series of 17 long peptides spanning LSA3-FL, to induce IL-10 production by lymphocytes isolated from volunteers of the clinical trial (Figure S3 in Supplementary Material). Simultaneously, spleen cells were cultured with LSA3-GP4 used as negative control, as this peptide did not induce IL-10 secretion in volunteers’ lymphocytes. The production of IL-10 was measured by ELISPOT. As shown in Figure 5, the peptides GP2 and/or GP8 induced IL-10 responses in LSA3-FL immunized mice engrafted with spleen cells derived from 3/5 of the tested donors as had been observed in human volunteers. These results confirm the presence of regulatory sequences in LSA3-FL regions that are absent in the short LSA3-729 construction, and which are detectable in the Hu-SPL-NSG model.

## Discussion

Humanized mouse models offer the potential of circumventing the current limitations of relevance of conventional rodent and other animal models. Efforts are being made to design humanized mouse models that can reliably be used in many disease areas and for a range of purposes; from the study of infection to pathogenesis, disease and immunogenicity. In the latter case, the ideal model could generate predictive proof-of-concept information in animal including characterization of immune responses induced by vaccine candidates, to advance candidates most likely to succeed into clinical development. Within this scope, the Hu-SPL-NSG model provides numerous advantages over current humanized mouse models.

*In vivo* studies of human immune responses to vaccines have been mostly modelized up to now in immunodeficient mice reconstituted with human peripheral blood mononuclear cells (Hu-PBL-SCID) (30–34). This approach is limited by the frequent occurrence of a graft versus host reaction (35–40), by the low survival of engrafted B lymphocytes and by the difficulty to raise primary immune responses [reviewed in Ref. (3–5)]. Alternatively, in other studies, mice were reconstituted with human CD34^+^ stem cells. This model ensures permanent colonization of the mouse immune system with human cells, however, it also has several limitations. Besides the ethical constraints and costs of stem cells, the T cells generated in this manner are not restricted to the human, but rather to the murine MHC (41). To overcome this limitation, models of SCID mice transgenic for a limited number of HLA alleles were developed and engrafted with CD34^+^ human cells. Despite an apparently satisfactory reconstitution of T and B cells, specific IgG responses remained of low magnitude and were inconsistently produced (42–44). Furthermore, IgM responses were of very high magnitude due probably to the fact that reconstituted B cells are of B1 like phenotype (43). An even more sophisticated model, the human bone marrow, liver, and thymus (BLT) mouse has been developed to study human immune responses to vaccines. Yet this model proved not able, despite its remarkable complexity, to produce neither specific IgG antibodies nor T cell responses even after repeated booster immunizations (45). Later on, HLA-A2 transgenic BLT mice immunized with BCG vaccine showed a moderate non-protective adaptive T cell immune response (46). *In vitro* models of immunization have also been developed; however, attempts to study the *in vitro* immune responses to vaccine candidates explored only the T cell component and not the antibody component of the immune response (47–52).

In the present study, we designed a new model, Hu-SPL-NSG in which human spleen cells are engrafted in immunodeficient NSG mice, and evaluated its relevance. To our knowledge, this model has not been previously employed for vaccine development. We selected the vaccine candidate LSA3-FL, a full length recombinant protein of the *P. falciparum* liver stage antigen 3 (LSA3) to determine whether the results obtained in the model would reflect those obtained during a Phase Ia clinical trial. The LSA3-FL malaria vaccine candidate proved to be poorly immunogenic in this clinical trial, despite previously demonstrating preclinical immunogenicity in a large variety of animal models including rats, mice of five breeds, macaques immunized using four distinct adjuvants, and aotus monkeys. This discrepancy provided a candidate vaccine fitted to assess the validity of the Hu-SPL-NSG model. It also led us to suspect then identify the presence of regulatory sequences in two regions of the full-length protein. In contrast, using the shorter LSA3-729 protein in previous experiments in apes, satisfactory B and T-cell responses were obtained with no indication for regulatory mechanisms. We therefore decided to compare in the Hu-SPL-NSG model the human immune response induced by the whole protein, LSA3-FL, with that induced by LSA3-729.

Our results showed that immunization of human splenic lymphocytes from five different donors with the full length version LSA3-FL failed to consistently induce the production of specific antibodies, or IFN-γ responses. These results are similar to those obtained in human volunteers immunized with LSA3-FL in whom weak antibody responses as well as an antigen-triggered decrease in IFN-γ responses were observed after the third immunization. The low immunogenicity of LSA3-FL is likely related to the presence of Treg epitopes, detected by expression of regulatory T cell markers. Indeed, immune responses in the Hu-SPL-NSG model were consistent with those observed using T cells from human volunteers immunized with LSA3-FL. Both in trial volunteers and in the Hu-SPL-NSG model, human lymphocytes produced IL-10 when stimulated *in vitro* with the synthetic peptides GP2 and GP8 corresponding, respectively, to the N-terminal and the central part of the molecule.

This situation differs from that observed using another vaccine candidate, MSP3, where amino-acid sequences having a downregulation effect were also present but could be readily detected in laboratory rodents, such as Balb/c and C57/Bl6 mice (53). Results obtained in the clinic with GMZ2, a MSP3-1-Glurp hybrid in which these regulatory regions have not been deleted suggest the same regulatory effect may take place in humans (54). Conversely, the Treg epitopes from LSA3 were not detected in rodent and primate species but were only revealed later, following clinical trial. All animal species immunized by LSA3-FL produced high B and T-cell immune responses, but only lymphocytes from volunteers of the clinical trial, and human cells in the Hu-SPL-NSG model, led to detect Treg responses.

Our results highlight several advantages of the Hu-SPL-NSG mouse, as compared to other models of human lymphocyte engraftment in immunocompromised mice, namely: (i) the reproducibility, since lymphocytes from each of five donors tested produced antibody and T cell responses, a reproducibility which is critical when it comes to formulation selection and is insufficient in several other models; individual variations were large but in fact similar to those found in the clinic from one volunteer to the other, (ii) the ability to generate an IgG antibody response (this study and unpublished data using other vaccine candidates) when other models are characterized by a dominance of IgM, (iii) the ability to generate antigen-specific IFN-γ responses, (iv) the ability to generate responses in various types of T cells, notably Th1 and Treg, which both bear a critical role in vaccine development, and (v) the very large numbers of cells of splenic origin, allowing to perform several detailed studies using the same well-characterized, cryopreserved sample in each.

These results underline the importance of human spleen as a source of immunocompetent cells. Indeed, the spleen is a secondary lymphoid organ that contains many discrete lymphocyte subsets, including mature lymphocytes and accessory cells, particularly mature dendritic cells in large numbers, which are best fitted to initiate human immune responses. As a consequence, the Hu-SPL-NSG model is relatively easy to establish and does not require the costly or time consuming phases that are necessary when hematopoietic precursors, fetal tissues, or PBMC, which require cell maturation, are the source of human cells. As large quantities of human cells can be harvested from the spleen, large series of animals can be engrafted, making it possible to compare the immunogenicity of several vaccine formulations upon lymphocytes of the same human source. This also permits a detailed analysis of immune responses, leading to identify and potentially remove Treg-inducing epitopes and select for T helper-inducing epitopes within a few weeks after the design of a new vaccine formulation.

In contrast to the results obtained with LSA3-FL, human splenocytes responded to immunization with LSA3-729 by producing high antibody titers as well as a strong IFN-γ T cell response. This is consistent with previous observations showing that several constructs covering the LSA3-729 region trigger antibody and IFN-γ producing T cell responses in Aotus monkeys (22) and chimpanzees (17), the latter being 99.4% homologous to human beings (55). Since a specific IFN-γ producing T cell response was observed in Hu-SPL-NSG immunized with LSA3-729, and not with LSA3-FL, the former appears to be a better vaccine candidate than the full length molecule. IFN-γ production has been associated with protection against experimental challenge by preerythrocytic stages of malaria parasites, as shown by a meta-analysis of protection obtained in chimpanzees, Aotus monkeys, or humans vaccinated with either LSA3-based formulations or irradiated sporozoites (26). Furthermore, taking advantage of an LSA3 orthologous gene expressed in *Plasmodium yoelii*, it was also possible to induce protection by the *P.f*-LSA3-729. In these experiments, protection was related to the ability of the animals to raise LSA3-specific Th1 cellular responses, with high IFN-γ secretion (18, 24). The role of CD4^+^ and/or CD8^+^ T cells as well as IFN-γ as important effectors of immunity against the preerythrocytic stages was described in various murine malaria studies [reviewed in Ref. (18, 56)]. Furthermore, it has been shown that IFN-γ can inhibit the *in vitro* development of *P. falciparum* liver forms in human hepatocytes (57) and that protection against preerythrocytic parasite development can be passively induced in animal models by injection of exogenous IFN-γ (58) or the related cytokine IL-12 (59).

Our results show that the Hu-SPL-NSG mouse constitutes a promising novel tool for preclinical vaccine development which could fill a major gap, even though it obviously requires further work. The variability in the response from one mouse to the other in the same experimental group was large, and is a limitation. This variability goes beyond the normal variations seen among inbred mice, or in clinical trials, and is possibly related to individual variability of lymphocytes survival in a heterologous host even without adaptive immune system and with altered innate defenses. Our future strategy is to address this question by analyzing the phenotype and distribution of the engrafted cell populations in the spleen of responder versus non-responder mice. Additional studies aimed at further improving the control of innate defenses are unquestionably needed. Other vaccine candidates having showed a major discrepancy between preclinical and clinical phases will be instrumental to continue to document the true potential of the model, e.g., in other fields such as viral or bacterial or therapeutic vaccines.

Laboratory animals, mostly mice, are the most widely used for preclinical assessment of vaccines, even though their relevance has yet to be established, likely because they are readily available. The Hu-SPL-NSG mouse model requires A3 animal house, sterile handling and is clearly more cumbersome and expensive than ordinary lab mice. Yet results show that it can predict events in humans and hence may deserve the additional effort. The Hu-SPL-NSG model presents, despite its limitations, an original, cost-effective, fast and above all predictive approach for improved screening of human immune responses to vaccine candidates prior to clinical trials. In this instance, had this model been available when the LSA3-FL clinical trial was decided, the results would have revealed the induction of strong Treg stimulation, and the candidate might not have progressed to the clinic. This would have not only saved a lot of time, efforts and money but would have also prevented the burden of immunization and *P. falciparum* mosquito challenge in human volunteers. Furthermore, it was the short segment, LSA3-729, originally identified by protective responses in humans that performed better. These results support the choice of LSA3-729 for clinical development of a second-generation LSA3-based vaccine.

## Ethics Statement

The human spleens were obtained from deceased organ transplant donors according to an ethical agreement with the National Organization for Organ & Tissues Donation & Transplantation (NOOTDT) in Lebanon. The informed consent to donate organs for transplantation or scientific research was signed by the patients themselves during their lifetime or by their parents following the death. Animal handling and experiments were conducted in accordance with US Guidelines for Experimental Animals.

## Author Contributions

HB-T and PD designed the study; SG performed the Hu-SPL-NSG experiments; EP produced the recombinant proteins and participated in primers design for RT-qPCR experiments; AS supervised spleen organ donation; SG, HB-T, PD, and SC analyzed the data; SG, HB-T, and PD wrote the manuscript.

## Conflict of Interest Statement

The authors declare that the research was conducted in the absence of any commercial or financial relationships that could be construed as a potential conflict of interest.

## Abbreviations

Hu-SPL-NSG, humanized-spleen cells-NOD-SCID-gamma null; LSA3-FL, liver stage antigen 3-full length; LSA3-729, liver stage antigen 3-729 (short form).
