# Supplementary material for: Evaluating Human Immune Responses for Vaccine Development in a Novel Human Spleen Cell-Engrafted NOD-SCID-IL2rγNull Mouse Model
Source: Front Immunol. 2018 Mar 23;9:601. doi: 10.3389/fimmu.2018.00601 (PMC5876497; doi:10.3389/fimmu.2018.00601)
Supplement: Supplementary file 1 [file image_1.pdf]

SI Fig 1: Example of Immune responses elicited in Animal Models immunized with LSA3 *Lactoccocus lactis* full lenght (LSA3-FL)

A. ELISA Antibodies to *Pf*LSA3

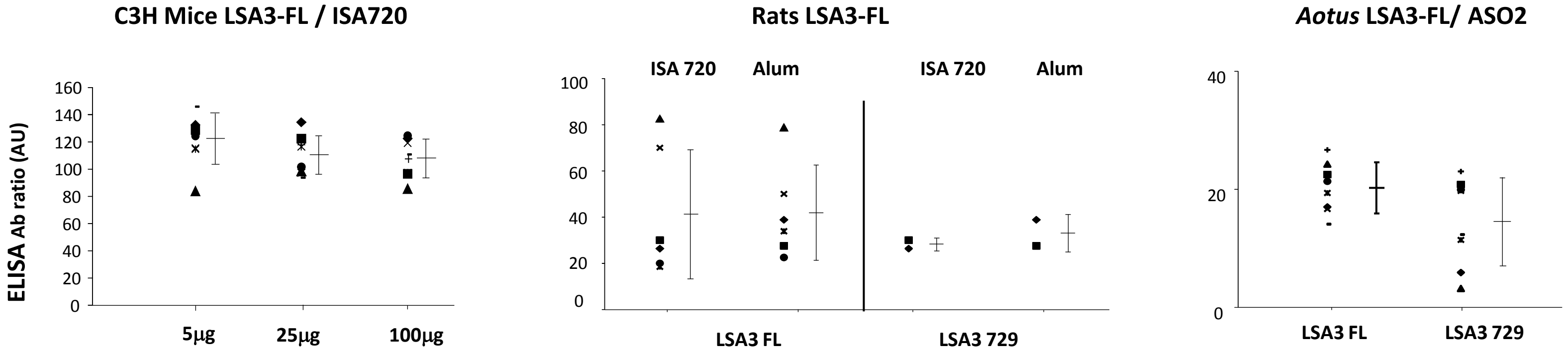

B. Ex- vivo IFN-γ to *Pf*LSA3 (ELIspot)

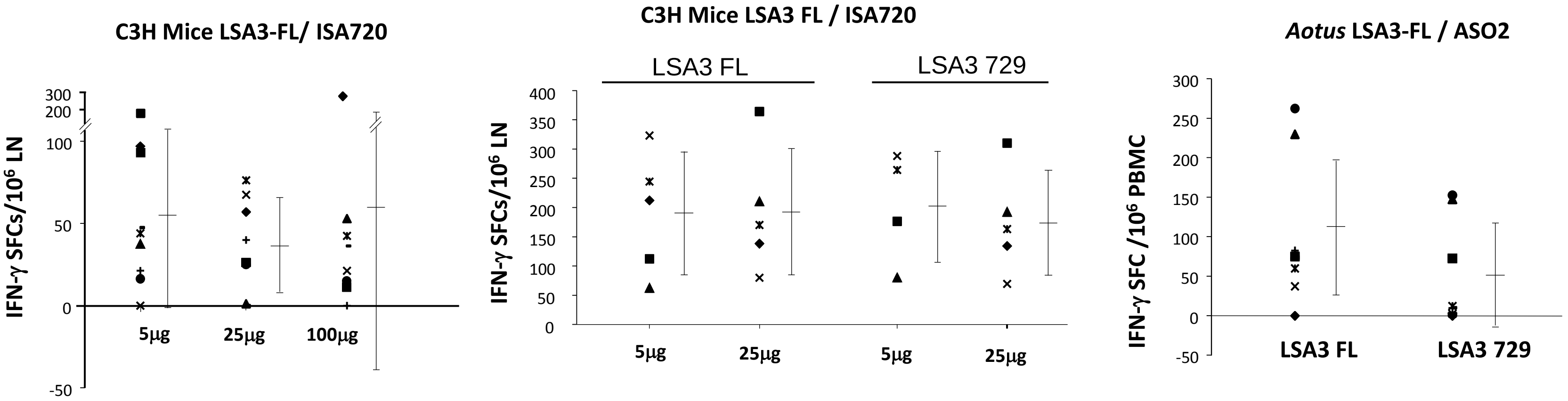

Methods are similar to those described in Perlaza BL. Et al Eur. J. Immunol. 2001. 31: 2200–2209, and in Perlaza et al Eur. J. Immunol. 2003. 33: 1321–1327, using for immunization , in mice or in Aotus monkeys, either the LSA3 Full length (LSAFL) or LSA3-DG 729 proteins indicated in those papers..
