# Supplementary material for: Evaluating Human Immune Responses for Vaccine Development in a Novel Human Spleen Cell-Engrafted NOD-SCID-IL2rγNull Mouse Model
Source: Front Immunol. 2018 Mar 23;9:601. doi: 10.3389/fimmu.2018.00601 (PMC5876497; doi:10.3389/fimmu.2018.00601)
Supplement: Supplementary file 2 [file image_2.pdf]

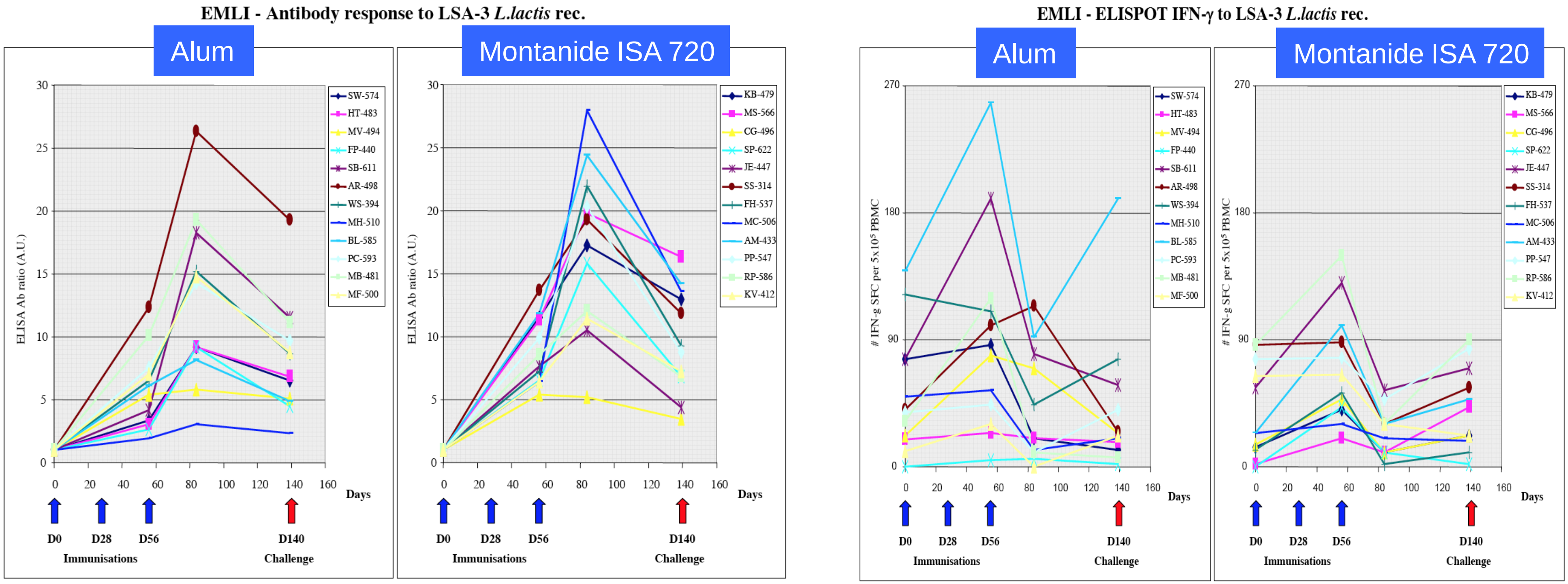

SI Fig 2 LSA-3-FL Antibody and IFN-gamma responses induced in human volunteers

ELISA results show a steady increase in titers after each immunization, according to expectations;
A relatively fast decrease in titer after the last immunization which is most unusual and had not been seen before in immunized mice, rats, rabbits, macaques, Aotus and chimpanzees, in whom titers remained very steady over time using a large variety of vaccine presentation methods.
ELISPOT show an expectable increase after the 1st and 2nd immunisations to relatively high levels of IFN-γ secretion.
The third immunization induces a major drop in the number of responding cells which is most unusual compared to eg chimpanzees and aotus.

Volunteers were immunized three times subcutaneously at 28-D intervals with 30 µg of a GMP-grade LSA-3-Full Length recombinant protein (PfLSA-3-rec-FL) expressed in Lactococcus lactis, and emulsified in either Aluminium hydroxide or Montanide ISA 720.
ELISPOT assays were conducted on fresh PBMCs collected at days 0, 56 (28 days after the second immunization), 84 (28 days after the third immunization), and 139 (one day before challenge) by in vitro stimulation with the PfLSA-3-rec protein Full length at 10 µg /ml.

PS: The clinical trial of LSA3-FL received approval from the Dutch National Ethical Committee and was conducted in compliance with ICH GCP and national clinical trial regulations, including obtaining signed informed consent from all subjects enrolled in the study. The trial was registered in the ClinicalTrials.gov database under the identifier NCT00509158
