# Supplementary material for: Evaluating Human Immune Responses for Vaccine Development in a Novel Human Spleen Cell-Engrafted NOD-SCID-IL2rγNull Mouse Model
Source: Front Immunol. 2018 Mar 23;9:601. doi: 10.3389/fimmu.2018.00601 (PMC5876497; doi:10.3389/fimmu.2018.00601)
Supplement: Supplementary file 3 [file image_3.pdf]

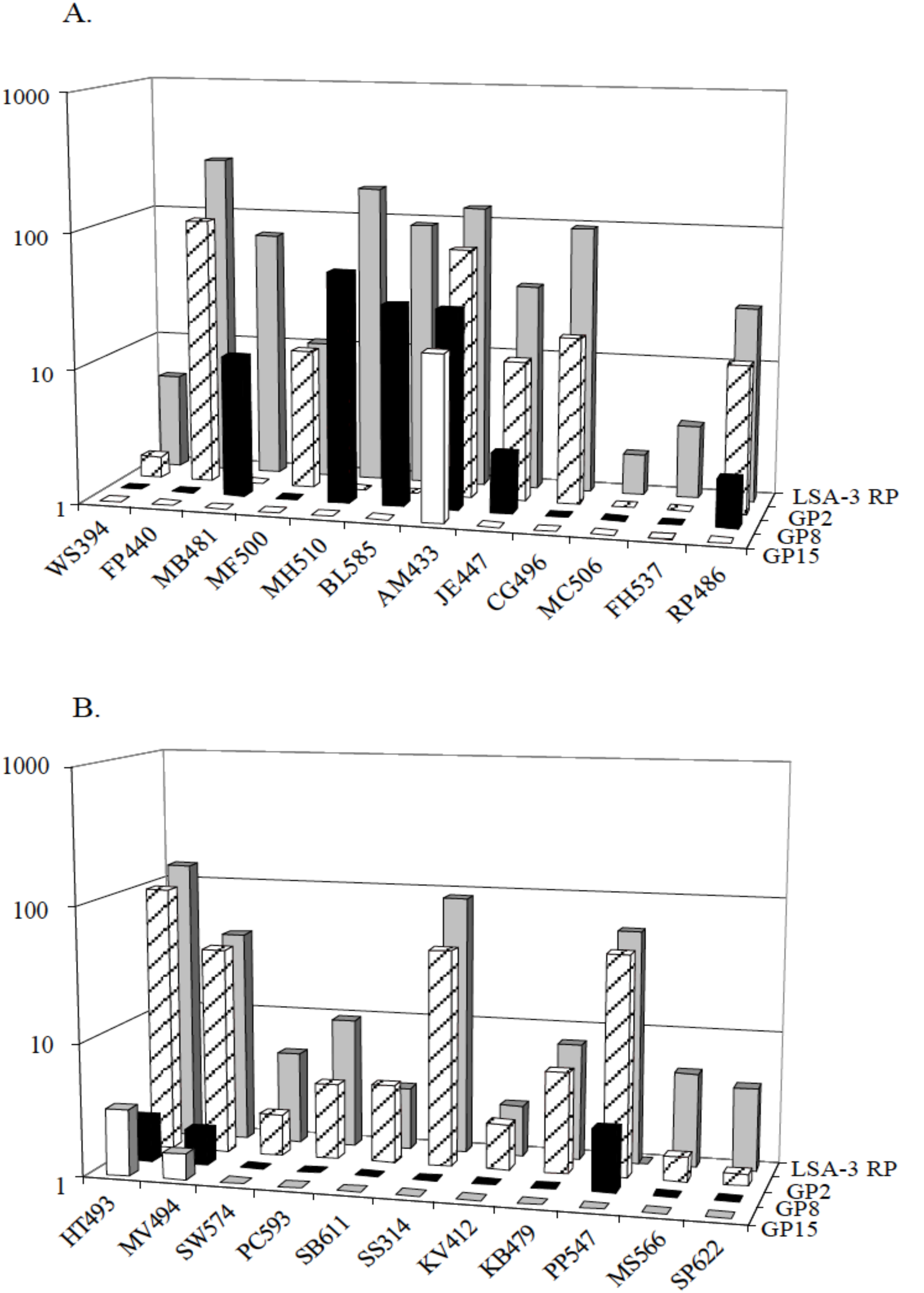

Supplementary Figure 3

Ex-vivo IL-10 response in volunteers immunized with Pf LSA-3 full length.
The cytokine response was assessed in PBMC collected at days 0, 56, and 84 from volunteers vaccinated with PfLSA-3 RP full length in alum (n=12), (A) or in Montanide ISA720 (n=11),(B).
Results with only 3 of 17 peptides are shown
IL-10 was titrated using a Cytokine Bead Array kit (BD) after 50h in vitro stimulation of the cells with the indicated antigens at 10µg/ml. For each volunteer at D84 data have been expressed as the ratio: ([IL-10 produced to antigen] - [IL-10 produced to medium])/[IL-10 produced to medium].

Immunological studies and investigations were conducted with the *Pf*LSA-3-rec protein together with a set of 17 overlapping long synthetic peptides (LSP), 60 to 185 amino acids in length, spanning the whole *P. falciparum* LSA3 protein. Phenotyping and cytokine profiling of LSA-3 specific T-cells was carried out with cryopreserved PBMC Cells that were stimulated with single antigens at the concentration of 10µg/ml in complete medium (RPMI 1640 supplemented with 2mM glutamine, antibiotics, Hepes, and 10% human AB serum). Cytokines levels were measured in supernatants from PBMC cultures stimulated for 50 to 60 h at 37°C with antigens at 10 µg/ml final concentration using the Cytokine Bead Array kit according to manufacturer's instructions (BD Biosciences) and read on FACSCalibur cytometer (BD). Supernatants were not diluted. Background levels were measured in supernatants from unstimulated PBMC cultures
