# Supplementary material for: Evaluating Human Immune Responses for Vaccine Development in a Novel Human Spleen Cell-Engrafted NOD-SCID-IL2rγNull Mouse Model
Source: Front Immunol. 2018 Mar 23;9:601. doi: 10.3389/fimmu.2018.00601 (PMC5876497; doi:10.3389/fimmu.2018.00601)
Supplement: Supplementary file 4 [file image_4.pdf]

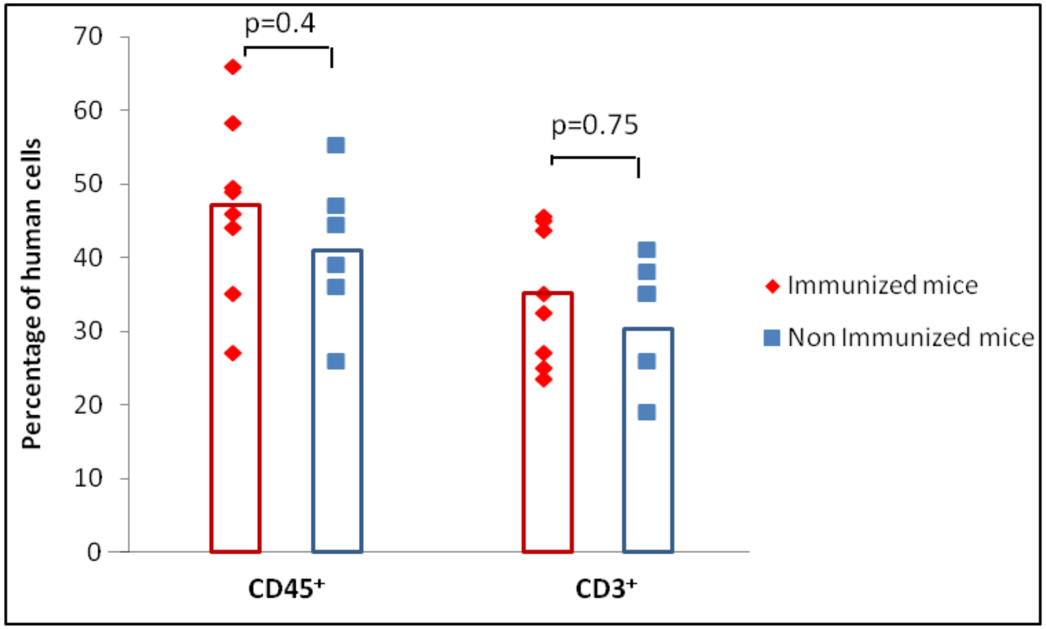

Supplementary Figure 4: Homing of human engrafted cells to the spleen of the Hu-SPL-NSG mice. Shown are the percentages of human CD45 and CD3 positive cells in the spleen organs of immunized and non immunized Hu-SPL-NSG mice. Spleen cell suspensions are incubated with mouse monoclonal antibodies specific for human surface antigens CD45 (leukocyte marker) or CD3 (T cell marker). The percentage of fluorescent human leukocytes is evaluated by fluorescence microscopy. Data are presented as mean values of results obtained with immunized and non immunized mice engrafted with human spleen cells from eight different donors of which 5 were used in this study. Each dot corresponds to the result obtained with spleen cells pooled from mice of one experimental group.
